# Supplementary material for: Brain virtual histology and volume measurement of a lizard species (Podarcis bocagei) using X-ray micro-tomography and deep-learning segmentation
Source: PeerJ. 2025 Sep 1;13:e19672. doi: 10.7717/peerj.19672 (PMC12422266; doi:10.7717/peerj.19672)
Supplement: Supplemental Information 2 — The volumes were measured from segmentations using deep-learning network and semi-automatic interpolation. [file peerj-13-19672-s002.docx]

| **Sample number** | **Olfactory [mm^3]** | **Telencephalon [mm^3]** | **Diencephalon [mm^3]** | **Midbrain [mm^3]** | **Cerebellum [mm^3]** | **Brainstem [mm^3]** |
| --- | --- | --- | --- | --- | --- | --- |
| 1 | 2.09 | 8.86 | 3.11 | 5.44 | 0.77 | 3.92 |
| 2 | 1.62 | 6.84 | 2.02 | 3.96 | 0.47 | 2.72 |
| 3 | 1.39 | 5.69 | 2.42 | 4.58 | 0.54 | 3.84 |
| 4 | 1.58 | 7.28 | 2.82 | 5.18 | 0.7 | 3.91 |
| 5 | 1.97 | 7.8 | 2.67 | 5.23 | 0.65 | 3.84 |
| 6 | 1.51 | 6.8 | 2.29 | 4.05 | 0.52 | 2.72 |
| 7 | 2.31 | 8.84 | 2.9 | 5.1 | 0.66 | 3.85 |
| 8 | 1.67 | 7.73 | 3.23 | 5.78 | 0.71 | 4.39 |
| 9 | 1.57 | 7.53 | 2.31 | 3.85 | 0.48 | 2.55 |
| 10 | 1.75 | 7.27 | 2.62 | 4.46 | 0.46 | 3.08 |
| 11 | 1.57 | 6.91 | 2.43 | 4.1 | 0.41 | 3.32 |
| 12 | 2.4 | 9.34 | 2.6 | 4.91 | 0.66 | 4.09 |
| 13 | 1.49 | 6.96 | 2.81 | 5.06 | 0.6 | 3.51 |
| 14 | 1.44 | 6.81 | 2.49 | 4.96 | 0.61 | 3.52 |
| 15 | 1.43 | 5.97 | 2.17 | 3.65 | 0.54 | 3.54 |
| 16 | 2.72 | 11.56 | 3.56 | 5.55 | 0.8 | 4.03 |
| 17 | 1.33 | 7.42 | 3 | 5.5 | 0.58 | 3.24 |
| 18 | 1.77 | 7.8 | 2.91 | 5.32 | 0.77 | 4.07 |
| 19 | 1.77 | 6.86 | 2.48 | 4.74 | 0.76 | 3.72 |
| 20 | 2.15 | 8.33 | 2.71 | 5.15 | 0.6 | 3.44 |
| 21 | 1.81 | 7.81 | 2.69 | 4.38 | 0.65 | 3.52 |
| 22 | 1.45 | 6.94 | 2.91 | 5.17 | 0.8 | 4.18 |
| 23 | 2.07 | 11.01 | 4.03 | 6.65 | 0.81 | 4.09 |
| 24 | 1.94 | 8.85 | 3.02 | 5.52 | 0.82 | 4.17 |
| 25 | 1.16 | 7.42 | 2.75 | 5.89 | 0.62 | 3.86 |
| 26 | 1.67 | 7.8 | 2.59 | 4.51 | 0.65 | 2.69 |
| 27 | 1.68 | 6.31 | 2.36 | 4.46 | 0.5 | 3.02 |
| 28 | 1.91 | 7.66 | 2.23 | 4.35 | 0.5 | 2.91 |
| 29 | 1.74 | 7.14 | 2.66 | 5.37 | 0.57 | 3.46 |
| **Mean** | 1.757241379 | 7.708275862 | 2.716896552 | 4.926551724 | 0.627931034 | 3.55862069 |
| **Standard Deviation** | 0.340596804 | 1.280824274 | 0.414138936 | 0.674553624 | 0.116334219 | 0.505826222 |
| **Standard Deviation in Percentage** | 19.38% | 16.62% | 15.24% | 13.69% | 18.53% | 14.21% |

**Supplementary Table S1.** The table depicts the raw data used to generate the values used in Figure 7. The volumes were measured from segmentations using deep-learning network and semi-automatic interpolation.
