# Supplementary material for: Brain virtual histology and volume measurement of a lizard species (Podarcis bocagei) using X-ray micro-tomography and deep-learning segmentation
Source: PeerJ. 2025 Sep 1;13:e19672. doi: 10.7717/peerj.19672 (PMC12422266; doi:10.7717/peerj.19672)
Supplement: Supplemental Information 3 [file peerj-13-19672-s003.docx]

**Supplementary Table S2.** The table depicts the dice score and relative error for Figure 8 for the two deep learning methods Biomedisa and AIMOS.

| Number of training dataset | Biomedisa | | AIMOS | |
| --- | --- | --- | --- | --- |
|  | DSC | RE (%) | DSC | RE (%) |
| 3 | 0.928 | 5.672 | 0.802 | 27 |
| 5 | 0.948 | 4.009 | 0.944 | 4.07 |
| 7 | 0.942 | 5.035 | 0.944 | 3.94 |
| 9 | 0.948 | 4.411 | 0.946 | 4.01 |
| 11 | 0.955 | 3.258 | 0.95 | 3.68 |
