# Supplementary figures and images for: Brain virtual histology and volume measurement of a lizard species (Podarcis bocagei) using X-ray micro-tomography and deep-learning segmentation
Source: PeerJ. 2025 Sep 1;13:e19672. doi: 10.7717/peerj.19672 (PMC12422266; doi:10.7717/peerj.19672)

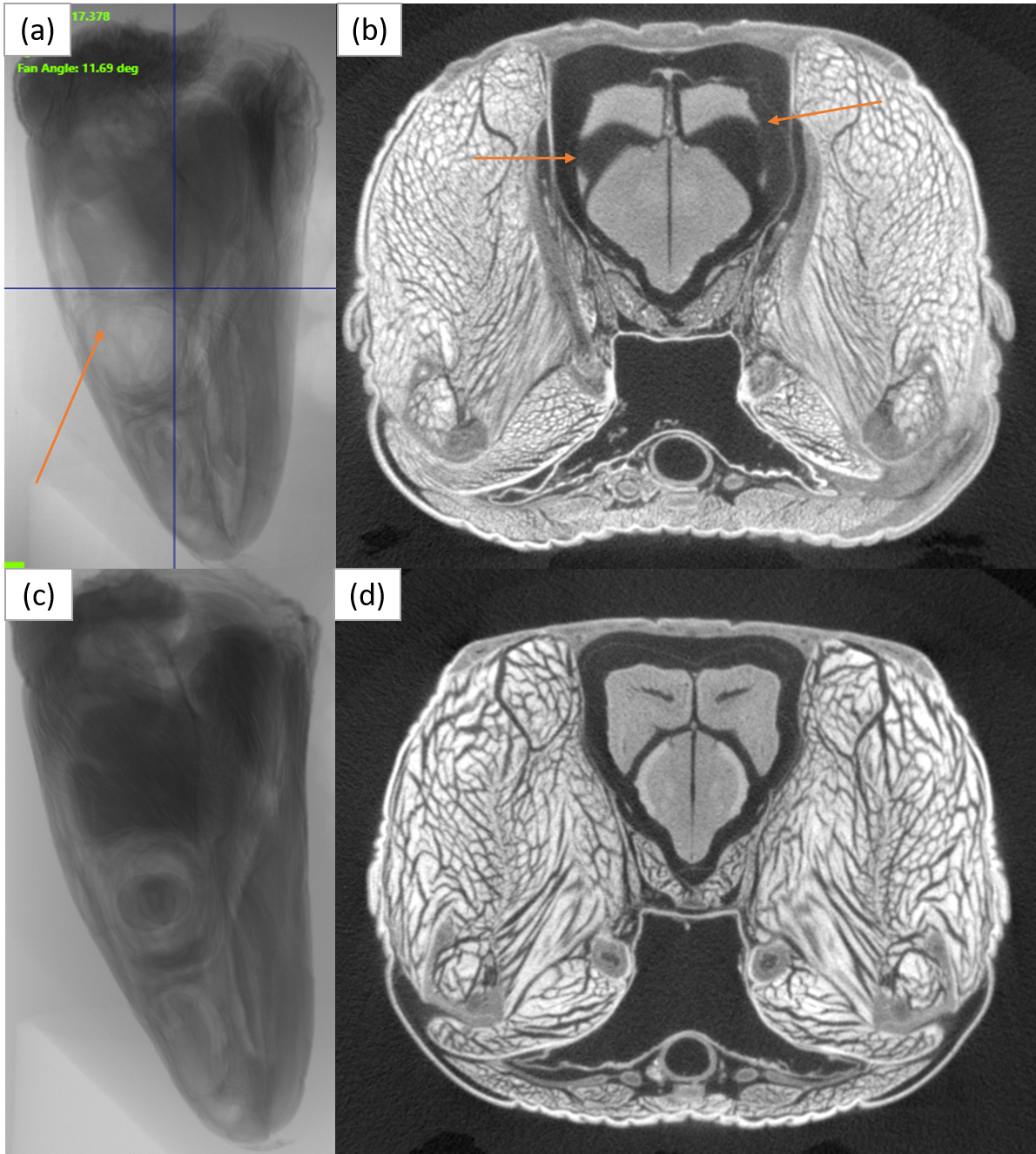

Supplement: Supplemental Information 4 — (a) An X-ray projection image of a sample, with the arrow indicating an area where the brain and eye were not fully stained. (b) A slice from the microCT reconstruction showing that the brain appears “missing” due to incomplete staining. (c, d) Examples of a well-stained sample for comparison. [file peerj-13-19672-s004.png]

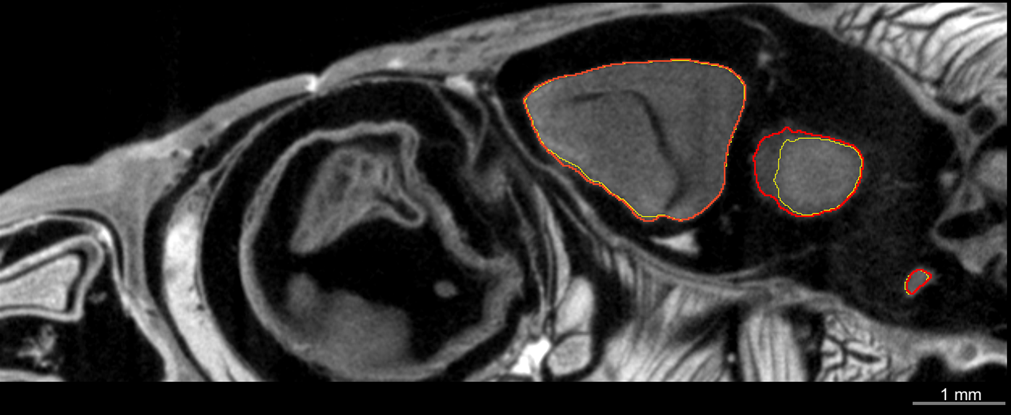

Supplement: Supplemental Information 5 [file peerj-13-19672-s005.png]
